# Low fruit consumption and folate deficiency are associated with LINE-1 hypomethylation in women of a cancer-free population

**DOI:** 10.1007/s12263-015-0480-4

**Published:** 2015-07-17

**Authors:** Antonella Agodi, Martina Barchitta, Annalisa Quattrocchi, Andrea Maugeri, Carolina Canto, Anna Elisa Marchese, Manlio Vinciguerra

**Affiliations:** Department of Medical and Surgical Sciences and Advanced Technologies “GF Ingrassia”, University of Catania, Via S. Sofia 87, 95121 Catania, Italy; LaPoSS, Laboratory of Policies and Social Services, University of Catania, Catania, Italy; Oncopath s.r.l, Floridia, SR Italy; UOC Laboratorio Analisi 1, P.O. “Vittorio Emanuele”, Catania, Italy; Institute for Liver and Digestive Health, University College London, Royal Free Campus, London, UK; Interdisciplinary Biomedical Research Centre, School of Science and Technology, Nottingham Trent University, Nottingham, UK

**Keywords:** Mediterranean diet, Folate intake, Epigenetics, Global methylation

## Abstract

Several dietary agents, such as micronutrient and non-nutrient components, the so-called bioactive food components, have been shown to display anticancer properties and influence genetic processes. The most common epigenetic change is DNA methylation. Hypomethylation of long interspersed elements (LINE-1) has been associated with an increased risk of several cancers, although conflicting findings have also been observed. The aim of the present study was to test the hypothesis that a low adherence to the Mediterranean diet (MD) and folate deficiency may cause LINE-1 hypomethylation in blood leukocytes of healthy women, and thus genomic instability. One hundred and seventy-seven non-pregnant women were enrolled. Mediterranean diet score (MDS) and folate intake were calculated using a food frequency questionnaire. LINE-1 methylation level was measured by pyrosequencing analysis in three CpG sites of LINE-1 promoter. According to MDS, only 9.6 % of subjects achieved a high adherence to MD. Taking into account the use of supplements, there was a high prevalence of folate deficiency (73.4 %). Women whose consumption of fruit was below the median value (i.e., <201 gr/day) were 3.7 times more likely to display LINE-1 hypomethylation than women whose consumption was above the median value (OR 3.7; 95 % CI 1.4–9.5). Similarly, women with folate deficiency were 3.6 times more likely to display LINE-1 hypomethylation than women with no folate deficiency (OR 3.6; 95 % CI 1.1–12.1). A dietary pattern characterized by low fruit consumption and folate deficiency is associated with LINE-1 hypomethylation and with cancer risk.

## Introduction

Emerging evidence suggests that the protective effect of nutrition can be mediated by reversible epigenetic mechanisms, representing an attractive target for cancer prevention (Su et al. 2012). Nutrients may induce transient or permanent alterations in the epigenetic marks that regulate the expression of genes involved in several processes and networks, which could be one of the factors leading to chronic diseases (Wilson 2008; Urdinguio et al. 2009). The most common epigenetic change is DNA methylation, which occurs in CpG islands and is often altered in cancer cells, which are characterized by both sporadic gene-specific hypermethylation and global DNA hypomethylation (Tollefsbol 2009). Furthermore, hypomethylation in repetitive elements, which enhances their activity as retrotransposons, has been suggested to have deleterious cell effects, through insertion, deletions and genomic rearrangements introducing genome instability (Kazazian 2004; Wallace et al. 2008). Particularly, long interspersed nucleotide elements 1 (LINE-1), considered the most common repetitive elements of interspersed DNA repeats (Weisenberger et al. 2005), are moderately CpG rich and most heavily methylated (Sheng et al. 2012). Lower level of LINE-1 DNA methylation in leukocytes has been associated with an increased risk of several cancers (Hsiung et al. 2007; Moore et al. 2008; Choi et al. 2009; Hou et al. 2010), and such evidences have been confirmed, also in tissues, by two previous meta-analyses (Woo and Kim 2012; Barchitta et al. 2014a). In addition, because of their high genome dissemination, LINE-1 methylation status has been proposed as a surrogate marker for estimating global DNA methylation level (Weisenberger et al. 2005; Yang et al. 2004). In women, changes in LINE-1 methylation levels in DNA from peripheral blood have been associated with cervical intraepithelial neoplasia and breast and bladder cancer risk (Piyathilake et al. 2011; DeRoo et al. 2014; Wilhelm et al. 2010).

Several dietary agents, such as micronutrient and non-nutrient components, the so-called bioactive food components, have been shown to display anticancer properties and influence genetic processes (Milner 2004; Ong et al. 2011; Hardy and Tollefsbol 2011). These components, constituents of several classes including folate, polyphenols, selenium, retinoids, fatty acids, isothiocyanates and allyl compounds, can affect DNA methylation through different mechanisms (Ong et al. 2011). Methyl groups for DNA methylation reactions are primarily supplied by choline and methionine and are regenerated endogenously by folate and vitamin B12 in the one-carbon metabolism pathway. Folate is a methyl donor in a number of molecular pathways (as DNA methylation, synthesis and repair) that are necessary for cellular replication and maintenance; thus, this vitamin is essential for fetal growth and development and for maternal well-being. Folate deficiency can lead to global hypomethylation inducing carcinogenesis at different sites (Kim 2007; Yang et al. 2009; Duthie 2011). In humans, dietary folate restriction or folic acid supplementation can alter DNA methylation (Jacob et al. 1998; Pufulete et al. 2005). However, some epidemiologic studies examining the association between dietary folate intake and leukocyte DNA methylation in healthy subjects reported null associations (Moore et al. 2008; Choi et al. 2009; Zhang et al. 2011a).

The Mediterranean diet (MD) is widely recognized to be the optimal diet for disease prevention and for good health, and independently of energy and macronutrient intakes, a better adherence to MD is associated with lower obesity risk (Mozaffarian et al. 2011; Martìnez-Gonzàlez et al. 2012; Barchitta et al. 2014b). Focusing on dietary patterns, a recent study reported that a high intake of dark green vegetables was associated with LINE-1 hypermethylation in a cancer-free population (Zhang et al. 2011a). Furthermore, an intervention study showed that subjects with greater adherence to MD had lower levels of LINE-1 methylation at the end of the study (Martín-Núñez et al. 2014).

This study is part of a larger project designed by our research group to investigate the relationships between diet, folate intake and nutritional status, and blood biomarkers in healthy women in Catania, Sicily.

The aim of the present study was to test the hypothesis that a low adherence to the MD and folate deficiency may cause LINE-1 hypomethylation in blood leukocytes of healthy women, and thus genomic instability and cancer risk.

## Methods

### Study design

During a three-year period (from 2010 to 2013), all consecutive non-pregnant healthy women referred to the Laboratory of the S. Bambino Hospital, Catania, Italy, an obstetric center for preconception, prenatal and/or postpartum care, were prospectively invited to participate in this cross-sectional study. The inclusion criteria were: (i) females aged between 13 and 50 years; (ii) non-pregnant; and (iii) no current or previous self-reported history of severe diseases including cancer. All eligible women were fully informed of the purpose and procedures of the study, and a signed written consent was obtained. The study protocol was approved by the ethics committee of the involved institution and performed according to the Declaration of Helsinki.

Data were collected by trained epidemiologists using a structured questionnaire to obtain information on sociodemographic and lifestyle data. Education level was collected, and women were classified into two categories: low (primary school, i.e., ≤8 years of school) and high (high school education or greater, i.e., >8 years of school) education level. Employment status was also recorded, and women were classified as employed or unemployed (including students and housewives). Body mass index (BMI) was calculated as weight (kg) divided by height (m^2^), based on criteria from the World Health Organization (World Health Organization 1995).

### Dietary assessment

Overall dietary intake and dietary folate intake were estimated by a validated semiquantitative food frequency questionnaire (FFQ) using the previous month as a reference period, as previously described (Agodi et al. 2011). For each of the food items, women were asked to report their frequency of consumption and portion size, trough indicative photograph atlas, to estimate the amount of each food item and to minimize inaccuracies.

Adherence to MD was assessed using the Mediterranean diet score (MDS), the nine-unit dietary score proposed by Trichopoulou (Trichopoulou et al. 1995) and revised by Couto et al. (2011). MDS components were calculated using median value as cutoff; thus, the score was population based. Through a categorical approach, women were classified into three groups, regarding adherence to MD. Particularly, MDS ≤25th percentile was defined as low adherence to MD (MDS 0–3); MDS >25th percentile but ≤90th percentile, as medium adherence to MD (MDS 4–6); and MDS >90th percentile, as high adherence to MD (MDS 7–9). Furthermore, adherence was redefined as follows: Women reporting a MDS ≤90th percentile of MDS distribution (MDS ≤ 6) were considered as with poor adherence to MD and the others as with high adherence (Barchitta et al. 2014b).

Folate and total caloric intakes were calculated using the USDA Nutrient Database (http://ndb.nal.usda.gov/) adapted to the Italian food consumption. Intake of folic acid from supplements was specifically addressed as previously described (Agodi et al. 2014a). Prevalence of folate deficiency was estimated by comparing folate intake with the Estimated Average Requirements (EAR) (Institute of Medicine Dietary Reference Intakes 2001).

### DNA extraction and methylation analysis

Genomic DNAs were extracted from whole blood using the Illustra blood genomic Prep Mini Spin Kit (GE Healthcare, Italy) according to the manufacturer’s protocol and stored at −20 °C. LINE-1 methylation level in lymphocytes was measured by pyrosequencing-based methylation analysis in three CpG sites of LINE-1 promoter (GenBank Accession No. X58075), after DNA bisulfite conversion using a previously reported protocol (Estécio et al. 2007; Piyathilake et al. 2012). The degree of methylation was expressed for each DNA locus as percentage of methylated cytosines over the sum of methylated and unmethylated cytosines. The degree of LINE-1 methylation was reported for each locus as well as the average percentage of methylation of the three evaluated CpG sites.

### Statistical analyses

Statistical analyses were performed using the SPSS software (version 22.0, SPSS, Chicago, IL). Descriptive statistics were used to characterize the population using frequencies, mean and standard deviation (SD) and median values.

Percentages of LINE-1 methylation levels were categorized based on tertile distribution. Low methylation (i.e., LINE-1 hypomethylation) was defined as LINE-1 methylation in the first tertile (T1) of the distribution, medium methylation was defined as LINE-1 methylation in the second tertile (T2) of the distribution, and high methylation level (i.e., LINE-1 hypermethylation) was defined as LINE-1 methylation in the third tertile (T3) of the distribution. Associations between LINE-1 hypomethylation and risk factors were assessed using those women in T3 of the distribution as reference group. The two-tailed Chi-squared test was used for the statistical comparison of proportions. In order to investigate associations between exposure to different factors and risk of hypomethylation, the crude odds ratios (ORs) and the corresponding 95 % confidence intervals (95 % CIs) were computed.

Continuous variables were tested using Student’s *t* test and one-way ANOVA. Correlations were assessed by Spearman’s rank correlations. Unconditional logistic regression analysis was used to separately evaluate the association between overall adherence to MD, as well as each food component, and LINE-1 methylation. The analyses were adjusted for age (>median vs. ≤median), education (low/medium vs. high school), nutritional status (overweight/obese vs. underweight/normal weight), smoking status (current smokers vs. non-smokers/former smokers) and total caloric intake (>median vs. ≤median).

The adjusted ORs with the respective CIs 95 % were reported. A *p* value < 0.05 was considered statistically significant in all performed analyses.

Given the lack of reference data regarding the association between folate deficiency and MD exposure and LINE-1 methylation levels, we assessed the statistical power and confidence interval (respectively, 80 and 95 %) at the end of the study (dos Santos Silva 1999) using EpiInfo version 6.

## Results

### Population characteristics

A total of 200 eligible women were asked to participate. Twenty-three women refused to participate, and thus 177 were included in the present analysis. The main characteristics of the population are given in Table 1.

**Table 1:** Characteristics of the study population (*n* = 177)

Characteristics	Mean (median)	*n* (%)
Age (years)	30 (28)	
BMI	24.3 (22.9)	
Nutritional status^a^		
Underweight		12 (6.8)
Normal weight		107 (60.4)
Overweight		32 (18.1)
Obese		26 (14.7)
Smoking status		
Current		41 (23.3)
Non-current		135 (76.7)
Employment status		
Employed		63 (35.6)
Unemployed		114 (64.4)
Education level		
Low		77 (43.5)
High		100 (56.5)
MDS	4.1 (4)	
Mediterranean diet adherence		
Low (MDS 0–3)		65 (36.7)
Medium (MDS 4–6)		95 (53.7)
High (MDS 7–9)		17 (9.6)
Food folate intake (µg/die)	245.8 (228.8)	
Dietary folate deficiency (cutoff 320 µg/day^b^)		
Yes		137 (77.4)
No		40 (22.6)
Supplement users		
Yes		12 (6.8)
No		165 (93.2)
Overall folate deficiency^c^		
Yes		130 (73.4)
No		47 (26.6)

*BMI* body mass index, *MDS* Mediterranean diet score

^a^Based on criteria from the World Health Organization (1995)

^b^Estimated Average Requirements by Institute of Medicine Dietary Reference Intakes (2001)

^c^Taking into account the use of supplements and Estimated Average Requirements by Institute of Medicine Dietary Reference Intakes (2001)

### Dietary assessment

The mean MDS value was 4.1 (median 4; range 0–9). According to MDS, only 9.6 % of subjects achieved a high adherence to MD. Adherence to MD was compared across baseline characteristics of enrolled women. Particularly, older women (>28 years old) reported a greater adherence to MD than the younger group (54.4 vs. 45.6 %; *p* = 0.016). Additionally, mean MDS was lower in obese women (3.2 vs. 4.3; *p* = 0.007), in those with low education level (3.8 vs. 4.4; *p* = 0.042), and in women who did not consume folic acid or vitamin supplements (3.9 vs. 5.3; *p* < 0.001) than in the others.

Mean folate intake was 245.8 µg/day (median 228.8 µg/day; range 46.3–773 µg/day). Only 6.8 % of women reported the use of folic acid supplements or of multimineral/multivitamin supplements containing folic acid. Taking into account the use of supplements, there was a high prevalence of folate deficiency (73.4 %). Notably, a higher proportion of women with folate deficiency were overweight/obese compared with women with no folate deficiency (41.5 vs. 8.5 %; *p* < 0.001). A higher proportion of women with poor adherence to MD were overweight/obese compared with women with high adherence to MD (76.9 vs. 41.2 %; OR 4.8; 95 % CI 1.7–13.3; *p* = 0.002).

### LINE-1 methylation analysis

Mean LINE-1 methylation level was 65.3 (SD: ± 3.3; range 52–74; all in 0–100 scale). LINE-1 methylation levels of tertiles were: T1: ≤64; T2: >64 to ≤ 66.3; and T3 > 66.3. Mean LINE-1 methylation levels for the three loci (80.1 ± 2.9; 57.8 ± 4.4; 62.9 ± 3.3) were positively correlated with each other (r_1−2_ = 0.357; r_1−3_ = 0.302; r_2−3_ = 0.838; *p* < 0.001).

Table 2 shows the comparison between tertiles distribution of LINE-1 methylation levels according to women characteristics. Women with folate deficiency had 3.1-fold increased risk to be hypomethylated, compared with women with no folate deficiency (T3 vs. T1: crude OR 3.1; 95 % CI 1.3–7.5), and the association showed a dose–response relationship (p-trend = 0.027). Although no significant difference was found considering overall MD adherence, when the nine Mediterranean food groups of the MDS were individually examined women whose consumption of fruit and nuts was below the median value (i.e., <201 gr/day) had 2.8-fold increased hypomethylation risk compared with women whose consumption was above the median value (T3 vs. T1: crude OR 2.8; 95 % CI 1.3–5.8). This association showed a dose–response relationship (p-trend = 0.022).

**Table 2:** Unadjusted univariate analysis of the association between LINE-1 methylation levels (tertiles distribution) and the main characteristics of the study population

Characteristics	Tertiles (T) distribution of LINE-1 methylation levels (range)			
	T1 (%)	T2 (%)	T3 (%)	p-trend^a^
Median age				
≤28	33 (53.2)	28 (49.1)	30 (51.7)	0.903
>28	29 (46.8)	29 (50.9)	28 (48.3)	
Age quartile distribution				
First quartile (13–23 years)	14 (22.6)	14 (24.6)	15 (25.9)	0.590
Second quartile (24–28 years)	16 (25.8)	10 (17.5)	14 (24.1)	
Third quartile (29–37 years)	11 (17.7)	16 (28.1)	8 (13.8)	
Fourth quartile (38–50 years)	21 (33.9)	17 (29.8)	21 (36.2)	
Body mass index (BMI)				
≥30	11 (17.7)	5 (8.8)	10 (17.2)	0.308
<30	51 (82.3)	52 (91.2)	48 (82.8)	
Nutritional status^b^				
Underweight	4 (6.5)	3 (5.3)	5 (8.6)	0.686
Normal weight	36 (58.1)	36 (63.2)	35 (60.3)	
Overweight	11 (17.7)	13 (22.8)	8 (13.8)	
Obese	11 (17.7)	5 (8.8)	10 (17.2)	
Education level				
Low	27 (43.5)	24 (42.1)	26 (44.8)	0.958
High	35 (56.5)	33 (57.9)	32 (55.2)	
Employment				
Employed	17 (27.4)	23 (40.4)	23 (39.7)	0.248
Unemployed	45 (72.6)	34 (59.6)	35 (60.3)	
Smoking status				
Current	14 (23.0)	9 (15.8)	18 (31.0)	0.154
Not current	47 (77.0)	48 (84.2)	40 (69.0)	
Supplement users				
Yes	4 (6.5)	3 (5.3)	5 (8.6)	0.768
No	58 (93.5)	54 (94.7)	53 (91.4)	
Overall folate deficiency^c^				
Yes	53 (85.5)	39 (68.4)	38 (65.6)	**0.027**
No	9 (14.5)	18 (31.6)	20 (42.6)	
Mediterranean diet adherence				
Poor adherence	57 (91.9)	52 (91.2)	51 (87.9)	0.733
High adherence	5 (8.1)	5 (8.8)	7 (12.1)	
Cereal intake (median value 101 gr/day)				
≤Median value	32 (51.6)	22 (38.6)	35 (60.3)	0.064
>Median value	30 (48.4)	35 (61.4)	23 (39.7)	
Vegetables intake (median value 263 gr/day)				
≤Median value	32 (51.6)	29 (50.9)	28 (48.3)	0.930
>Median value	30 (48.4)	28 (49.1)	30 (51.7)	
Fruit and nuts intake (median value 201 gr/day)				
≤Median value	39 (62.9)	27 (47.4)	22 (37.9)	**0.022**
>Median value	23 (37.1)	30 (52.6)	36 (62.1)	
Meat intake (median value 82 gr/day)				
>Median value	29 (46.8)	26 (45.6)	34 (58.6)	0.299
≤Median value	33 (53.2)	31 (54.4)	24 (41.4)	
Dairy intake (median value 213 gr/day)				
>Median value	33 (53.2)	29 (50.9)	27 (46.6)	0.761
≤Median value	29 (46.8)	28 (49.1)	31 (53.4)	
Fish intake (median value 24 gr/day)				
≤Median value	37 (59.7)	25 (43.9)	27 (46.6)	0.178
>Median value	25 (40.3)	32 (56.1)	31 (53.4)	
Legumes intake (median value 27 gr/day)				
≤Median value	30 (48.4)	27 (47.4)	32 (55.2)	0.658
>Median value	32 (51.6)	30 (52.6)	26 (44.8)	
Unsaturated/saturated ratio intake (median value 2.4)				
≤Median value	27 (45.5)	33 (57.9)	30 (51.7)	0.291
>Median value	35 (56.5)	24 (42.1)	28 (48.3)	
Alcohol intake				
Abstainers or >25 gr/day	54 (87.1)	53 (93.0)	46 (79.3)	0.099
Between 5 and 25 gr/day	8 (12.9)	4 (7.0)	12 (20.7)	

^a^Statistically significant *p* values (*p* < 0.05) are indicated in bold font

^b^Based on criteria from the World Health Organization (1995)

^c^Taking into account the use of supplements and Estimated Average Requirements by Institute of Medicine Dietary Reference Intakes (2001)

**Fig. 1:**
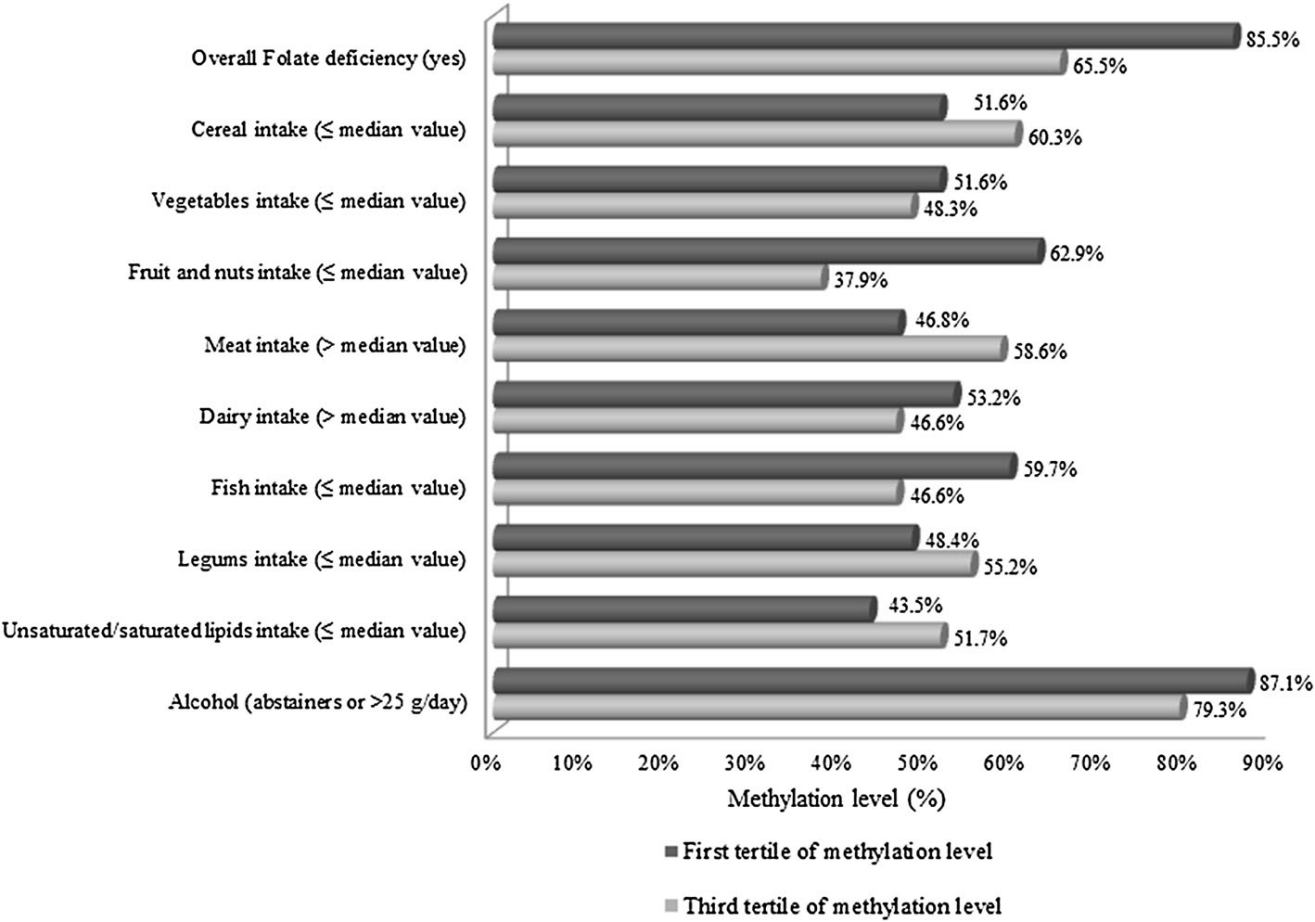
Variables included in the logistic regression analysis for hypomethylation levels. Adjusted for age, smoking status, daily energy intake, nutritional status and education level

The results of the regression analysis (Fig. 1) adjusting for the main confounders confirmed that women with folate deficiency were significantly more likely to show LINE-1 hypomethylation than women with adequate folate intake (OR 3.6; 95 % CI 1.0–12.1; *p* = 0.04). Considering the nine Mediterranean food groups of the MDS, women with lower fruit and nuts intake (i.e., below the median value, 201 gr/day) reported 3.7-fold increased hypomethylation risk compared with women with higher intake (OR 3.7; 95 % CI 1.4–9.9; *p* = 0.01).

## Discussion

Benefits of high adherence to MD and of adequate folate intake are reported in the general population (Sofi et al. 2010; Couto et al. 2011; Tamura and Picciano 2006), and particularly in women of childbearing age, because of the effect of diet on their health and well-being, on pregnancy outcome, and on newborns’ health (Tamura and Picciano 2006; Barchitta et al. 2014b; Olmedo-Requena et al. 2013). Besides, recent studies provide evidence for a beneficial role of the MD on endometrial (Filomeno et al. 2015) and breast cancer risk (Buckland et al. 2013; Castellò et al. 2014). Women of fertile age, often more willing to adhere to recommendations involving lifestyle change, represent a good target for preventive nutritional intervention (Olmedo-Requena et al. 2013). In our population, adherence to MD was poor, with 36.7 % of women reporting a MDS below or equal to 3, similarly to the percentage described from Italian women of the EPIC population (34.1 %) (Couto et al. 2011). These findings confirm that, in recent years, all over the world and in particular in the Mediterranean countries, the diet is shifting away from the traditional MD pattern. This is probably due to the globalization process, characterized by cultural and social changes, which is likely to have an impact on changes in food habits (da Silva et al. 2009; Couto et al. 2011), also in Sicily. In addition, in our study, approximately 30 % of women were overweight/obese confirming previously reported results in Italian populations (Gallus et al. 2013; Barchitta et al. 2014b). Thus, strategies to promote MD and weight control are needed for disease prevention. Besides, in Italy, where no folic acid fortification has been introduced but only supplementation in the periconception period is recommended, a high prevalence of folate deficiency was previously described (Pounis et al. 2014; Agodi et al. 2011, 2013). Particularly, it has recently reported that only 7.4 % of Italian women of childbearing age exceeded the optimal dietary folate intake of 400 µg/day, which is quite safe at a reproductive age (Pounis et al. 2014). In the present study, 73.4 % of women were folate deficient and, thus, public health strategies to increase folate intake are needed.

There are growing interests in determining how dietary patterns and specific micronutrients and macronutrients may affect global DNA methylation levels. In our study, we hypothesized that a low adherence to MD and folate deficiency cause LINE-1 hypomethylation, a biomarker for cancer risk. Particularly, it has been reported that a higher degree of LINE-1 methylation in blood was associated with a lower risk of cervical intraepithelial neoplasia (Piyathilake et al. 2011). Moreover, LINE-1 hypomethylation in DNA from peripheral blood was associated with an increased risk of breast cancer (DeRoo et al. 2014) and bladder cancer in women (Wilhelm et al. 2010). Finally, global DNA methylation and LINE-1 methylation levels have been suggested as markers of inherited breast cancer susceptibility (Wu et al. 2011; Delgado-Cruzata et al. 2014).

The mean LINE-1 methylation level did not differ according to age, socioeconomic factors, smoking and nutritional status. However, current studies report discordant findings (Zhang et al. 2011a, b; Zhu et al. 2012; Gomes et al. 2012). No association was found between poor adherence to MD and LINE-1 methylation level. The lack of association might be caused by the intrinsic limits of the method adopted to assess MDS. In fact, the summation of equally weighted dietary component scores implies that each component is equally important and additively related to health (Moeller et al. 2007). Indeed, when all the nine Mediterranean food components were individually examined by DNA methylation level, women with low fruit are more likely to be hypomethylated. Remarkably, this amount (i.e., 201 gr/day) represents little more than one serving a day of fruit, which is certainly below the recommended levels (Ministry of Health 2003). A prudent dietary pattern, characterized by a high intake of vegetables and fruits, has been previously associated with lower prevalence of leukocyte LINE-1 hypomethylation in a cancer-free population, in a dose-dependent manner (Zhang et al. 2011a). Similarly, in a population of women at higher risk of developing cervical precancer or cancer, women with the healthiest dietary pattern were 3.3 times more likely to have higher leukocyte LINE-1 methylation than women with the unhealthiest one (Piyathilake et al. 2012). A recent trial has been conducted in order to explore the associations between changes in lifestyle modifications, such as diet, and global epigenetic biomarkers in blood of overweight female breast cancer survivors. After a dietary change and physical activity weight loss intervention, LINE-1 methylation levels were significantly elevated compared to baseline. Particularly, a 10 % increase in the frequency of fruit consumption was associated with an increase in LINE-1 methylation levels of 0.42 % (Delgado-Cruzata et al. 2015). Finally, in a prospective cohort intervention study, subjects with a lower adherence to MD showed higher values of LINE-1 methylation one year later the nutritional intervention (Martín–Núñez et al. 2014). Although the above-mentioned studies highlighted the beneficial effects of a healthy dietary pattern on LINE-1 methylation, to the best of our knowledge, the present study is the first observational study, conducted to investigate the hypothesis of a possible association between low adherence to MD and DNA hypomethylation in a cancer-free population (Agodi et al. 2014b).

Notably, in the present study, women with folate deficiency showed increased risk of hypomethylation than the others. Folate is necessary in DNA methylation, and epidemiological studies have suggested that this vitamin might alter DNA methylation levels, but findings are conflicting (Jacob et al. 1998; Pufulete et al. 2005; Moore et al. 2008; Choi et al. 2009; Zhang et al. 2011a, b). It has been suggested that low folate status, as well as inadequate amounts of other methyl donors, is associated with decreases in global DNA methylation, and thus with an increased risk of cancer (Piyathilake et al. 2011; Agodi et al. 2014b; Davis and Uthus 2004; Crider et al. 2012). On the contrary, in a cross-sectional study conducted in healthy Japanese women, a higher folate intake level has been associated with a lower global methylation level of leukocyte DNA, measured by luminometric methylation assay, LUMA (Ono et al. 2012). Furthermore, dietary intake of folate from fortified foods has been positively correlated with LINE-1 methylation in a cancer-free population (Zhang et al. 2012).

High consumption of fruit and nuts may provide multiple bioactive components, such as one-carbon nutrients, including folate, and antioxidants, which may interact in the prevention of DNA hypomethylation. Since sources of folate naturally occur in a wide variety of foods, as those included in the traditional MD, intervention on the overall dietary pattern rather than on single nutrients or food groups may be a more effective way to protect against cancer risk through dietary epigenetic regulation (Zhang et al. 2011a, b).

Our study has some limitations. The cross-sectional design of the study does not allow determination of causality. Secondly, we have not measured serum folate to support our hypothesis; folate intake was estimated by a FFQ. The use of a FFQ does not preclude measurement errors and may suffer from inaccuracies of volunteers’ recall. Nevertheless, the present FFQ was specifically developed for the use among our population and was previously validated against a 4-day weighted dietary record, with a correlation coefficient in accordance with other FFQ validation studies (Agodi et al. 2011). Furthermore, the relationship between dietary patterns and DNA methylation may be confounded by genetic polymorphisms, not evaluated in the present study. Particularly, the genetic polymorphism C677T in the methylene tetrahydrofolate reductase (MTHFR) may result in global DNA hypomethylation through an interaction with folate status (Friso et al. 2002). *MTHFR* C677T mutation shows a frequency of approximately 20 % in Sicily, Italy (Wilcken et al. 2003; Agodi et al. 2011). Since CT and TT subjects need a higher supply of folic acid, LINE-1 methylation levels might not reflect the same condition in all subjects. Besides, this study was not designed to measure other important protective micronutrients or other specific methyl donors, such as other B-vitamins (Piyathilake et al. 2011) that were not estimated in the present analysis.

Sample size calculations based on power (dos Santos Silva 1999) showed that the study has enough power (80 %) to detect statistically significant results for multiple exposure–outcome relationships, such as for low fruit intake and folate deficiency. However, in order to assess the relationship between poor adherence to MD and LINE-1 hypomethylation, the study population should be increased.

In conclusion, results from the present study provide a strong indication that a dietary pattern characterized by low fruit consumption and folate deficiency is associated with LINE-1 hypomethylation and with cancer risk. A recent meta-analysis added new evidence to the growing literature on the role of LINE-1 hypomethylation in cancer and shows that LINE-1 methylation levels are significantly lower in certain cancer types compared to healthy controls (Barchitta et al. 2014a). Future large-scale studies are needed to evaluate whether the association between dietary pattern and DNA methylation holds longitudinally. Leukocyte LINE-1 methylation may serve as a biomarker for dietary interventions designed to reduce the risk of cancerous and precancerous conditions. Therefore, intervening to change the unhealthiest dietary patterns in favor of the healthiest options among women may reduce the risk of hypomethylation and, consequently, of cancer (Wentzensen et al. 2009; Lim et al. 2008).

## References

[CR1] Agodi A (2011). Increase in the prevalence of the MTHFR 677 TT polymorphism in women born since 1959: potential implications for folate requirements. Eur J Clin Nutr.

[CR2] Agodi A (2013). Folate dietary intake and blood biomarkers reveal high risk groups in a Mediterranean population of healthy women of childbearing potential. Ann Nutr Metab.

[CR3] Agodi A, Barchitta M, Quattrocchi A, Marchese AE, Boffetta P (2014). Folate deficiency is not associated with increased mitochondrial genomic instability: results from dietary intake and lymphocytic mtDNA 4977-bp deletion in healthy young women in Italy. Mutagenesis.

[CR4] Agodi A et al (2014b) Diet, genetic and epigenetic signatures in women of childbearing age from a Mediterranean population: perspectives for public health. In: Abstracts of the 2014 Conference of the International Society of Environmental Epidemiology (ISEE). Abstract 2093. Research Triangle Park, NC: Environmental Health Perspectives

[CR5] Barchitta M, Quattrocchi A, Maugeri A, Vinciguerra M, Agodi A (2014). LINE-1 Hypomethylation in Blood and Tissue Samples as an Epigenetic Marker for Cancer Risk: a Systematic Review and Meta-Analysis. PLoS ONE.

[CR6] Barchitta M, Quattrocchi A, Adornetto V, Marchese AE, Agodi A (2014b) Tumor necrosis factor-alpha -308 G > A polymorphism, adherence to Mediterranean diet, and risk of overweight/obesity in young women. Biomed Res Int 742620 10.1155/2014/742620 PMC4083707 25028665

[CR7] Buckland G (2013). Adherence to the Mediterranean diet and risk of breast cancer in the European prospective investigation into cancer and nutrition cohort study. Int J Cancer.

[CR8] Castellò A (2014). Spanish Mediterranean diet and other dietary patterns and breast cancer risk: case-control EpiGEICAM study. Br J Cancer.

[CR9] Choi JY (2009). Association between global DNA hypomethylation in leukocytes and risk of breast cancer. Carcinogenesis.

[CR10] Couto E (2011). Mediterranean dietary pattern and cancer risk in the EPIC cohort. Br J Cancer.

[CR11] Crider KS, Yang TP, Berry RJ, Bailey LB (2012). Folate and DNA Methylation: review of Molecular Mechanisms and the Evidence for Folate’s Role. Adv Nutr..

[CR12] da Silva R (2009). Worldwide variation of adherence to the Mediterranean diet, in 1961–1965 and 2000–2003. Public Health Nutr..

[CR13] Davis CD, Uthus EO (2004). DNA methylation, cancer susceptibility, and nutrient interactions. Exp Biol Med (Maywood)..

[CR14] Delgado-Cruzata L (2014). Differences in DNA methylation by extent of breast cancer family history in unaffected women. Epigenetics.

[CR15] Delgado-Cruzata L (2015). Dietary modifications, weight loss, and changes in metabolic markers affect global DNA methylation in Hispanic, African American, and Afro-Caribbean breast cancer survivors. J Nutr.

[CR16] DeRoo LA (2014). Global DNA methylation and one-carbon metabolism gene polymorphisms and the risk of breast cancer in the Sister Study. Carcinogenesis.

[CR17] dos Santos Silva I (1999). Cancer epidemiology: principles and methods.

[CR18] Duthie S (2011). Folate and cancer: how DNA damage, repair and methylation impact on colon carcinogenesis. J Inherited Metab Dis..

[CR19] Estécio MR (2007). LINE-1 hypomethylation in cancer is highly variable and inversely correlated with microsatellite instability. PLoS ONE.

[CR20] Filomeno M (2015). Mediterranean diet and risk of endometrial cancer: a pooled analysis of three Italian case-control studies. Br J Cancer.

[CR21] Friso S (2002). A common mutation in the 5,10-methylenetetrahydrofolate reductase gene affects genomic DNA methylation through an interaction with folate status. Proc Natl Acad Sci USA.

[CR22] Gallus S (2013). Overweight and obesity prevalence and determinants in Italy: an update to 2010. Eur J Nutr.

[CR23] Gomes MV (2012). Age-related changes in the global DNA methylation profile of leukocytes are linked to nutrition but are not associated with the MTHFR C677T genotype or to functional capacities. PLoS ONE.

[CR24] Hardy TM, Tollefsbol TO (2011). Epigenetic diet: impact on the epigenome and cancer. Epigenomics..

[CR25] Hou L (2010). Blood leukocyte DNA hypomethylation and gastric cancer risk in a high-risk Polish population. Int J Cancer.

[CR26] Hsiung DT (2007). Global DNA methylation level in whole blood as a biomarker in head and neck squamous cell carcinoma. Cancer Epidemiol Biomarkers Prev..

[CR27] Institute of Medicine Dietary Reference Intakes (2001). Food and Nutrition Board.

[CR28] Jacob RA (1998). Moderate folate depletion increases plasma homocysteine and decreases lymphocyte DNA methylation in postmenopausal women. J Nutr.

[CR29] Kazazian HH (2004). Mobile elements: drivers of genome evolution. Science.

[CR30] Kim YI (2007). Folate and colorectal cancer: an evidence-based critical review. Mol Nutr Food Res.

[CR31] Lim U (2008). Genomic methylation of leukocyte DNA in relation to colorectal adenoma among asymptomatic women. Gastroenterology.

[CR32] Martìnez-Gonzàlez MA (2012). A 14-Item Mediterranean Diet Assessment Tool and Obesity Indexes among High-Risk Subjects: the PREDIMED Trial. PLoS ONE.

[CR33] Martín-Núñez GM (2014). Methylation levels of the SCD1 gene promoter and LINE-1 repeat region are associated with weight change: an intervention study. Mol Nutr Food Res.

[CR34] Milner JA (2004). Molecular targets for bioactive food components. J Nutr.

[CR35] Ministry of Health (2003) Linee guida per una sana alimentazione italiana, 2003. Available at: http://www.salute.gov.it/imgs/C_17_pubblicazioni_652_allegato.pdf. Accessed 4 Oct 2014

[CR36] Moeller SM (2007). Dietary patterns: challenges and opportunities in dietary patterns research: An Experimental Biology Workshop, April 1, 2006. J Am DietAssoc..

[CR37] Moore LE (2008). Genomic DNA hypomethylation as a biomarker for bladder cancer susceptibility in the Spanish Bladder Cancer Study: a case-control study. Lancet Oncol..

[CR38] Mozaffarian D, Hao T, Rimm EB, Willett WC, Hu FB (2011). Changes in diet and lifestyle and long-term weight gain in women and men. N Engl J Med.

[CR39] Olmedo-Requena R (2013). Factors associated with a low adherence to a Mediterranean diet pattern in healthy Spanish women before pregnancy. Public Health Nutr.

[CR40] Ong TP, Moreno FS, Ross SA (2011). Targeting the epigenome with bioactive food components for cancer prevention. J Nutrigenet Nutrigenomics..

[CR41] Ono (2012). Association of dietary and genetic factors related to one-carbon metabolism with global methylation level of leukocyte DNA. Cancer Sci.

[CR42] Piyathilake CJ (2011). A higher degree of LINE-1 methylation in peripheral blood mononuclear cells, a one-carbon nutrient related epigenetic alteration, is associated with a lower risk of developing cervical intraepithelial neoplasia. Nutrition..

[CR43] Piyathilake CJ (2012). A dietary pattern associated with LINE-1 methylation alters the risk of developing cervical intraepithelial neoplasia. Cancer Prev Res (Phila)..

[CR44] Pounis G (2014). Folate intake and folate serum levels in men and women from two European populations: the IMMIDIET project. Nutrition.

[CR45] Pufulete M (2005). Effect of folic acid supplementation on genomic DNA methylation in patients with colorectal adenoma. Gut.

[CR46] Sheng W (2012). LINE-1 methylation status and its association with tetralogy of fallot in infants. BMC Med Genomics.

[CR47] Sofi F, Abbate R, Gensini GF, Casini A (2010). Accruing evidence on benefits of adherence to the Mediterranean diet on health: an updated systematic review and meta-analysis. Am J Clin Nutr.

[CR48] Su LJ, Mahabir S, Ellison GL, McGuinn LA, Reid BC (2012). Epigenetic Contributions to the Relationship between Cancer and Dietary Intake of Nutrients, Bioactive Food Components, and Environmental Toxicants. Front Genet..

[CR49] Tamura T, Picciano MF (2006). Folate and human reproduction. Am J Clin Nutr.

[CR50] Tollefsbol TO (2009) Role of Epigenetics in Cancer. In: Tollesfbol TO (ed) Cancer Epigenetics

[CR51] Trichopoulou A (1995). Diet and overall survival in elderly people. BMJ.

[CR52] Urdinguio RG, Sanchez-Mut JV, Esteller M (2009). Epigenetic mechanisms in neurological diseases: genes, syndromes, and therapies. Lancet Neurol.

[CR53] USDA Nutrient Database. Available at: http://ndb.nal.usda.gov/. Accessed 4 Oct 2014

[CR54] Wallace NA, Belancio VP, Deininger PL (2008). L1 mobile element expression causes multiple types of toxicity. Gene.

[CR55] Weisenberger DJ (2005). Analysis of repetitive element DNA methylation by MethyLight. Nucleic Acids Res.

[CR56] Wentzensen N, Sherman ME, Schiffman M, Wang S (2009). Utility of methylation markers in cervical cancer early detection: appraisal of the state-of-the-science. Gynecol Oncol.

[CR57] Wilcken B (2003). Geographical and ethnic variation of the 677C > T allele of 5,10 methylenetetrahydrofolate reductase (MTHFR): findings from over 7000 newborns from 16 areas worldwide. J Med Genet.

[CR58] Wilhelm CS (2010). Implications of LINE1 Methylation for Bladder Cancer Risk in Women. Clin Cancer Res.

[CR59] Wilson AG (2008). Epigenetic regulation of gene expression in the inflammatory response and relevance to common diseases. J Periodontol.

[CR60] Woo HD, Kim J (2012). Global DNA Hypomethylation in Peripheral Blood Leukocytes as a Biomarker for Cancer Risk: a Meta-Analysis. PLoS ONE.

[CR61] World Health Organization (1995). Physical status: the use and interpretation of anthropometry. Report of a WHO Expert Committee. World Health Organ Tech Rep Ser.

[CR62] Wu HC (2011). Global DNA methylation levels in girls with and without a family history of breast cancer. Epigenetics..

[CR63] Yang AS (2004). A simple method for estimating global DNA methylation using bisulfite PCR of repetitive DNA elements. Nucleic Acids Res.

[CR64] Yang Q, Bostick RM, Friedman JM, Flanders WD (2009). Serum folate and cancer mortality among U.S. adults: findings from the third national health and nutritional examination survey linked mortality file. Cancer Epidemiol Biomark Prev.

[CR65] Zhang FF (2011). Dietary patterns are associated with levels of global genomic DNA methylation in a cancer-free population. J Nutr.

[CR66] Zhang FF (2011). Physical activity and global genomic DNA methylation in a cancer-free population. Epigenetics..

[CR67] Zhang FF (2012). White blood cell global methylation and IL-6 promoter methylation in association with diet and lifestyle risk factors in a cancer-free population. Epigenetics..

[CR68] Zhu ZZ (2012). Predictors of global methylation levels in blood DNA of healthy subjects: a combined analysis. Int J Epidemiol.

